# Variation in blood pressure control and blood biochemistry between patients attending early vs. late hemodialysis in Bahrain

**DOI:** 10.1186/1753-6561-9-S1-A11

**Published:** 2015-01-14

**Authors:** MAM Ebrahim, HAM Radhi, MA Almuqamam, D Whitford

**Affiliations:** 1 Royal College of Surgeons in Ireland, Medical University of Bahrain, Al Sayh, Bahrain; 2 Department of Family and Community Medicine RCSI Bahrain, Adliya, Bahrain

## Background

The Coordination of biological rhythms with medical treatment is termed Chronotherapy. This therapy utilizes the use of circadian or other rhythmic cycles of the human body in the optimization of treatment. This study examines blood pressure control and serum biochemistry level in early vs. late hemodialysis patients; attempting to identify a significant chronological variance. The parameters explored were chosen due to their tremendous effects on morbidity and mortality of hemodialysis patients. As these patients are amongst the highest risk groups in developing cardiac events; enhancing blood pressure(BP)control is a major contributor to their survival and well-being.

## Methods

This is a cross-sectional study looking at hemodialysis patients in two main hemodialysis centers in Bahrain.The size of the study is determined by the total number of patients who agree to participate.Currently the total number of patients registered is approximately 350.The sample size needed for a 95%confidence interval of +/-2 is 306. Patients in these centers are divided into three time-shifts.First batch (A) starts from 7AM -11AM,the second (B) from 1PM – 5PM and the third (C) from 6PM – 10PM.Patients’ age, sex, timing of dialysis, Calcium, Phosphorus, Urea, Creatinine, Bicarbonate, Parathyroid hormone, Hemoglobin, Albumin, Alkaline phosphatase (ALP), Diabetes Mellitus status and BP control were obtained from the patient’s files and the Ministry of Health RFW system. Formal statistical analysis using SPSS was applied with a statistical significance set at a p-value of <0.05.

## Results

A total of 329 patients were included, group A had 106 patients, group B had 135 whereas group C had 88 patients. Males made up 51.4% of the total sample while females constituted 48.6%. Out of all serum biochemistry parameters, five were found of statistically significant variance between the three groups. Average serum Urea, Creatinine and ALP were found to be lowest in group C measuring at (18.7,(95% 16.9-20.4),P-value 0.04), (672.5,(95% 612.2-732.7),P-value 0.001) and (132.4,(95% 118.3-146.5),P-value 0.032) respectively. While average Albumin&HCO3 were highest in group A measuring at (33.1,(95% 32.3-33.9),P-value 0.002) and (18.2,(95% 17.5-18.8),P-value 0.01) respectively. BP control did not achieve statistically significant difference among the three groups but when compared between AM hours (7-11AM) and PM hours (1-10PM),results showed better control in AM shift attenders (53.8%, P-value 0.18).

## Conclusions

Late attenders had a better serum Urea,Creatince&ALP levels while early attenders had better HCO3&Albumin.However, BP control showed no statistical significance in the original setting of the study. Physicians can apply these results in their practice by placing patients to the appropriate time-shifts as suggested by their blood studies.

